# Geographic variation in women’s empowerment: a multilevel analysis of India’s National Family Health Survey 2021

**DOI:** 10.7189/jogh.15.04159

**Published:** 2025-06-27

**Authors:** Yun-Jung Eom, S V Subramanian, Rockli Kim

**Affiliations:** 1 Interdisciplinary Program in Precision Public Health, Department of Public Health Sciences, Graduate School of Korea University, Seoul, South Korea; 2 Harvard Center for Population and Development Studies, Cambridge, Massachusetts, USA; 3 Department of Social and Behavioral Sciences, Harvard T. H. Chan School of Public Health, Boston, Massachusetts, USA; 4 Division of Health Policy and Management, College of Health Science, Korea University, Seoul, South Korea

## Abstract

**Background:**

Women’s empowerment is vital for sustainable development, yet gender inequality persists across India’s sociocultural and geographic landscape. Most initiatives in India focus on state- and district-level frameworks, failing to address the localised challenges at smaller geographic levels.

**Methods:**

This study quantified and visualised community variation in women’s empowerment using the fifth National Family Health Survey (2019–2021). Women’s empowerment was assessed across three domains – attitudes toward violence, social independence, and decision-making – using the globally validated Survey-based Women’s emPowERment index (SWPER). Four-level logistic regression models were used to partition the geographic variation in women’s empowerment into state, district, and community levels, and produce precision-weighted estimates.

**Results:**

The final sample included 76 683 women, 9104 communities, 720 districts, and 36 states across India. Communities contributed most to the total geographic variation in attitude to violence (47.1%) and decision-making (69.6%), while states were the largest contributors in social independence (57.7%). Geographically, under-empowered communities were concentrated in the south for attitude to violence; in the north for social independence; and widely dispersed for decision-making.

**Conclusions:**

Strategies to improve women’s empowerment in India should span across multiple geographic units while integrating domain-specific approaches to facilitate meaningful and sustainable progress.

Women’s empowerment is well recognised as both a moral imperative and a strategic cornerstone for human development [1,2]. Women’s active participation in school, the workforce, and health care is essential for economic growth and social progress [3,4], reinforced by the United States (UN)’s inclusion of gender equality as the fifth Sustainable Development Goal (SDG) [5]. Most recently, however, a report from UN Women revealed significant setbacks in achieving gender equality, urging accelerated action to shift the current trajectory [6]. The report reveals concerning statistics: one in eight women and girls experienced sexual and/or physical violence from an intimate partner in 2023, and 18.7% of women were married before age 18, with child marriage projected to persist until 2092 if current trends continue [6]. The report highlights the delays in implementing gender-responsive laws and policies, alongside challenges in measuring the progress of gender equality due to insufficient data on women’s experiences.

India is no exception to these global challenges. The country’s traditional patriarchal structure has historically perpetuated male dominance across social, legal, political, and economic spheres [7]. Despite efforts by the Indian government to enhance women’s access to socioeconomic resources [8], India ranked 129th among 146 countries in the Global Gender Gap Index [9], displaying severe gender disparities in various sectors. For example, while education has seen some improvements since the implementation of The Right to Education Act in 2009, a gender gap in this area remains; women are lagging behind men by roughly 17% in literacy rates [9], and by 10% in the proportion of completing 10 or more years of schooling as of 2021 [10]. Widespread violence and harmful practices against women also persist in India [11]. Over 40% of Indian men reportedly justify wife-beating under certain situations [12], and nearly one-third of ever-married Indian women have experienced any form of violence [13]. Although the rate of girls’ child marriage has declined over the past decade, regional disparities remain, with central and eastern regions showing higher concentrations of early marriage and motherhood [14].

Most initiatives aimed at improving women’s empowerment in India have been designed for and implemented at the state and district levels. States serve as the primary macro-policy units, while districts function as subdivisions within states, acting as central hubs for programme development, administration, and implementation [15]. For instance, the Beti Bachao Beti Padhao (Save the Daughter, Educate the Daughter), launched in 2015, focuses on declining child sex ratio and promoting girls’ education, especially in districts with a low child sex ratio [16]. The Janani Suraksha Yojana (Scheme for the Safety of Mothers) has incentivised institutional deliveries among pregnant women from low-income families through district-level public health facilities since 2005 [17]. Additionally, Mahila-E-Haat (Women’s E-Market) supports women entrepreneurs by providing an online marketing platform [18], and Swadhar Greh (A Home for Rehabilitation for Destitute Women) addresses the needs of women in distress by offering shelter, food, and rehabilitation services [19], both being implemented at state and district levels. Notably, many policies and programmes in India have largely focused on the economic empowerment of women, and less so on altering women’s gendered attitudes or enhancing their decision-making power.

At the same time, women’s empowerment and gender norms may exhibit substantial geographic heterogeneity at multiple scales [20,21]. Even within the same district or state, community-level disparities in women’s empowerment can arise from differences in local infrastructure, governance capacity, and social support systems. Rural communities, for instance, often face infrastructural deficiencies that limit women’s access to education and economic opportunities. Additionally, variability in local leadership, policy implementation, and the presence of community-based women’s organisations can create distinct environments for empowerment, with some communities benefiting from female support networks and advocacy initiatives while others lack such resources. Though not explored in terms of women’s empowerment, previous studies based on India have highlighted considerable differences in health and development outcomes across communities within the same district or state, underscoring the need for localised approaches [22–30].

Given the potential variation in women’s empowerment across smaller geographic levels in India, it becomes essential to analyse women’s empowerment at multiple geographical units. A few existing studies on geographic patterns of women’s empowerment in India focused on state-level variation [8,11,31], often overlooking inequalities at smaller levels. Furthermore, they were confined to using unvalidated measures, though they employed various indicators to capture the multidimensionality of women’s empowerment. This study addresses these gaps by using the globally validated Survey-based Women’s emPowERment index (SWPER) [32]. The SWPER assesses women’s empowerment across three domains: attitude to violence indicating whether women justify wife-beating under different situations; social independence indicating women’s preconditions for self-sufficiency (*e.g*. education), and decision-making indicating women’s autonomy within the household. As each domain of SWPER captures distinct aspects of women’s empowerment, applying this measure across different geographic levels of India may contribute to a comprehensive understanding of women's roles and agency in India.

Specifically, the current study has three aims. First, we partition the total geographic variation in women’s empowerment across states, districts, and communities in India for each domain – attitude to violence, social independence, and decision-making – to assess the relative importance of macro- and micro-geographic units. Second, we examine the within-state and within-district variation in women’s empowerment across the three domains based on the precision-weighted estimates of women’s empowerment. Lastly, we geo-visualise the distinct patterns of clustering of under-empowered communities for each domain to identify high-priority regions for intervention. By adopting this approach, the study seeks to provide actionable insights for optimising resource allocation and tailoring interventions to enhance women’s empowerment in India.

## METHODS

### Data source

We utilised nationally representative data from the fifth National Family Health Survey (NFHS-5) (17 June 2019 to 30 April 2021) that covers all 707 districts nested within 36 states or Union Territories (UTs) in India. The NFHS collects a wide range of information related to population and health as part of the Demographic and Health Surveys (DHS) [33]. The NFHS was designed to select primary sampling units or clusters – villages in rural areas and census enumeration blocks in urban areas (hereafter collectively labelled as communities) – using probability proportionate to population size from districts within states. Survey interviews were conducted by trained field teams recruited by designated Field Agencies in each state, considering educational background and relevant qualifications, with female and male interviewers assigned to respondents of the same sex. Before each interview, informed consent was obtained from all participants to ensure ethical compliance. To maintain data quality, the survey employed close fieldwork supervision across five adjacent districts at a time and adhered to standardised manuals to ensure uniform procedures across states. Further details on the data collection and sampling procedures can be found in the latest NFHS report [34].

### District geometry

India’s sub-national geography is constantly updating due to administrative reasons, with changes in district borders and geographies. For example, there were 640 districts at the time of NFHS-4 and 707 districts at the time of NFHS-5. For this study, we used an updated geometry of 720 districts instead of the 707 districts since the state of Andhra Pradesh (AP) created 13 new districts in April 2022 [35]. Incorporating these additional districts is essential, as none of the districts from AP listed in NFHS-5 align with the updated district boundaries of that state, hence precluding any substantive interpretations of women’s empowerment within AP.

To update the district geography from AP, Assembly Constituency (AC) boundaries were first linked to the 707 district boundaries from the DHS Spatial Data Repository [36] using the linkage information from the Chief Electoral Officer of AP [35]. Each state legislature elects representatives from ACs, and district boundaries for each state contain the boundaries of these ACs. Hence, the AC shapefiles in AP can create updated district boundaries by merging the AC polygons to establish the new district boundaries [35]. After adjusting the district shapefile to have the same external boundary of India aligning with the Survey of India’s specifications [37], the new 720 districts for NFHS-5 were created. As district reorganisations continue in India, future research can adopt a similar method to integrate updated boundaries and maintain consistency in longitudinal analyses.

### Study population

Of 724 115 women aged 15–49 years in the NFHS-5 data set, we excluded women not currently married or in union (n = 211 707) since it was a precondition of women for most items included in SWPER. We also excluded women initially not selected for question modules on the attitude to violence and the decision-making domain (n = 435 498), resulting in 76 910 eligible women. After excluding missing data on items of SWPER (n = 227), the final analytic sample resulted in 76 683 women aged 15–49 years from 9104 communities, 720 districts, and 36 state/UTs (Figure S1 in the **Online Supplementary Document**).

This study was exempt from ethical approval (KUIRB-2023-0245-01) as it utilised secondary analysis of publicly available data. We also followed the Strengthening the Reporting of Observational Studies in Epidemiology (STROBE) reporting guideline.

### Primary outcome

We used the SWPER index, a globally validated measure of women’s empowerment that encompasses three domains: attitude to violence, social independence, and decision-making [32]. In the attitude to violence domain, five items assessing women’s attitudes on whether a husband beating his wife is justified under different situations (*e.g*. wife goes out without telling husband, wife argues with husband) were included. In the social independence domain, six items representing women’s prerequisites for women’s empowerment (*e.g*. women’s educational attainment, age at first marriage, and frequency of reading newspapers or magazines) were included. In the decision-making domain, three items reflecting women’s involvement in decisions under specific settings (*e.g*. women’s health care and large household purchases) were included.

Following the coding strategy outlined by Ewerling et al. (2020), we extracted these 14 items from NFHS-5 and computed the scores of each domain, where a higher score indicates higher women’s empowerment (Table S1 in the **Online Supplementary Document**). The scores for each domain were standardised and subsequently categorised into terciles (high, medium, and low) following the approach proposed by prior SWPER-based research [38,39]. Then, for ease of interpretation, we constructed a binary variable where women’s low level of empowerment is coded as 1, a primary outcome of our study, whereas high and medium women’s empowerment are together coded as 0.

### Statistical analyses

Our final analytic sample followed a hierarchical structure of women i (level 1), nested within communities j (level 2), districts k (level 3), and states l (level 4). Given the nested structure of our sample, we adopted a 4-level logistic regression model specified as:

logit(Y_ijkl_) = β_0_ + (u_0jkl_ + v_0kl_ + f_0l_)

where *Y*_ijkl_ is the log odds of low level of empowerment for women *i*, *β_0_* is the constant, and *u*_0jkl_, *v*_0kl_, and *v*_0kl_are the residuals for communities *j*, districts *k*, and states *l*, respectively. Residuals were assumed to be normally distributed with a mean of 0 and a variance of *u*_0jkl_  *~ N*(0, σ^2^_u0_), *v*_0kl_  *~ N*(0, σ^2^_v0_), and *v*_0kl_  *~ N*(0, σ^2^_f0_). The term σ^2^_u0_ represents within-district, between-community variance; σ^2^_v0_ is within-state, between-district variance, and σ^2^_f0_ is between-state variance. The variance partitioning coefficient (VPC) for each level was computed to identify the relative importance of geographic levels by dividing the variance of a given level by the total geographic variance:


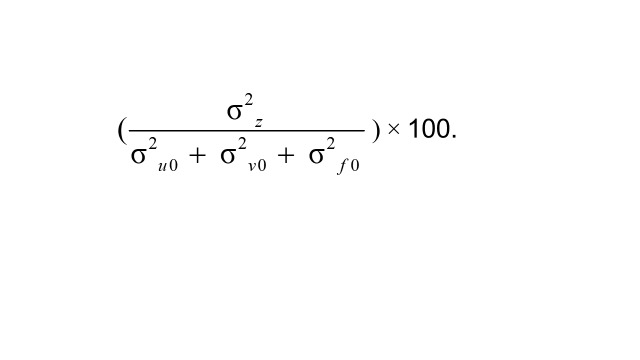
) × 100.

Since we used a binary outcome, level-1 variance is not freely estimated. For all multilevel analyses, Markov Chain Monte Carlo (MCMC) methods were employed following a Bayesian approach where prior information is used to maximise a likelihood function [40]. Priors were calculated using Iterative Generalized Least Squares with a 2nd order predictive quasi-likelihood approximation [41]. Markov Chain Monte Carlo methods with Bayesian estimation were chosen to ensure more accurate and well-calibrated interval estimates for random-effects variances in three-level logistic regression models [42], while maintaining methodological consistency with previous studies on small area variation estimation [22,25,43]. For the stability of estimates, we specified a burn-in of 500 cycles and monitoring of 5000 iterations of chains.

Using the residuals derived in the 4-level model above, precision-weighted estimates of women’s low level of empowerment were computed for each community as:


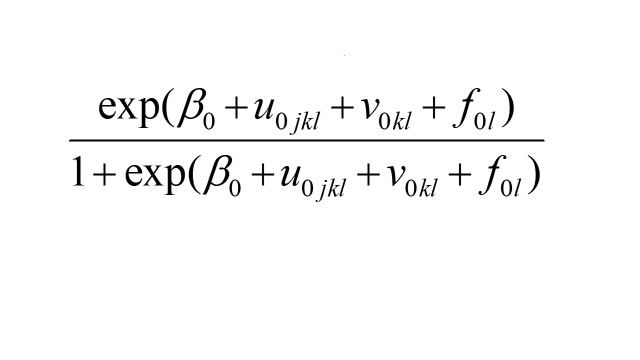
.

Precision-weighted estimation is known to down-weigh or shrink the influence of clusters with lower reliability towards the overall mean [44]. Based on these precision-weighted estimates, descriptive analyses and visual inspections were performed. First, we calculated the mean prevalence for each state based on precision-based estimates. We also examined the extent of within-state community variation through box plots. Second, we estimated the mean prevalence for each district and assessed the within-district community variation by computing the standard deviation (SD) for each district. Then, the correlation coefficients between the district mean prevalence and their within-district community variation were computed. Finally, we produced maps to visually identify the geographic patterns of clustering of under-empowered communities, defined as communities with a prevalence of women’s low level of empowerment equal to or higher than the national mean. For maps, we used equal intervals as cut-offs with ten categories.

All analyses were conducted for each domain of women’s empowerment. Multilevel modeling was performed using Stata, version 16.0 (StataCorp LLC, College Station, TX, USA) and MLwiN, version 3.05 (Centre for Multilevel Modelling, University of Bristol, Bristol, UK) with *runmlwin* command [45]. All maps were generated using *R*, version 4.2.2 (R foundation for Statistical Computing, Vienna, Austria).

## RESULTS

**Figure 1:**
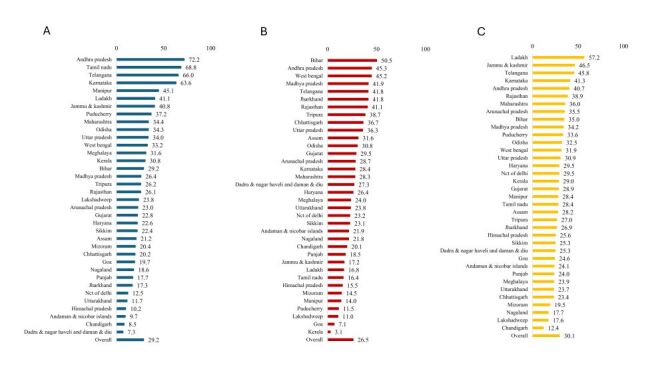
Precision-weighted prevalence (%) of women’s low level of empowerment by 36 states or Union Territories in India. **Panel A**. Attitude to violence. **Panel B**. Social independence. **Panel C**. Decision-making. Prevalence of women’s low level of empowerment by states was based on cluster-level precision-based estimates.

The final analytic sample consisted of 76 683 women aged 15–49 years from 9104 communities, 720 districts, and 36 states (Figure S1 and Table S2 in the **Online Supplementary Document**). Across states, 29.2% of women had low level of empowerment in the attitude to violence domain, 26.5% in the social independence domain, and 30.1% in the decision-making domain (**Figure 1**). For each state, the prevalence of women’s low level of empowerment substantially varied across the 3 domains. For example, Tamil Nadu in the southern region performed poorly in the attitude to violence (ranked 2nd), but relatively well in the social independence (ranked 29th) and the decision-making domains (ranked 20th). Few states consistently performed poorly across all three domains of women’s empowerment, such as Andhra Pradesh and Telangana.

### Relative importance of geographic levels

When partitioning the total geographic variation of women’s low level of empowerment to each level, different patterns were observed across three domains. In the attitude to violence domain, communities contributed the most to the total geographic variation (47.1%), followed by states (41.7%) and districts (11.3%) (**Table 1**). On the contrary, in the social independence domain, states were the largest contributor to geographic variation (57.7%), while communities were the second largest (27.3%), and districts were the least (15.0%). Lastly, in the decision-making domain, communities mostly accounted for the largest variation (69.6%), with only a small gap of variation between states (16.0%) and districts (14.4%).

**Table 1:** Four-level variance component model for women’s low level of empowerment in India

Variables	Variance estimate (95% CI)	VPC (%)
**Attitude to violence**		
State	0.86 (0.52–1.41)	41.7
District	0.23 (0.19–0.28)	11.3
Community	0.97 (0.92–1.04)	47.1
**Social independence**		
State	0.57 (0.34–0.94)	57.7
District	0.15 (0.12–0.17)	15.0
Community	0.27 (0.24–0.30)	27.3
**Decision-making**		
State	0.15 (0.08–0.26)	16.0
District	0.13 (0.11–0.16)	14.4
Community	0.64 (0.60–0.69)	69.6

CI – confidence interval, VPC – variance partitioning coefficient

### Within-state community variation in women’s low level of empowerment

**Figure 2:**
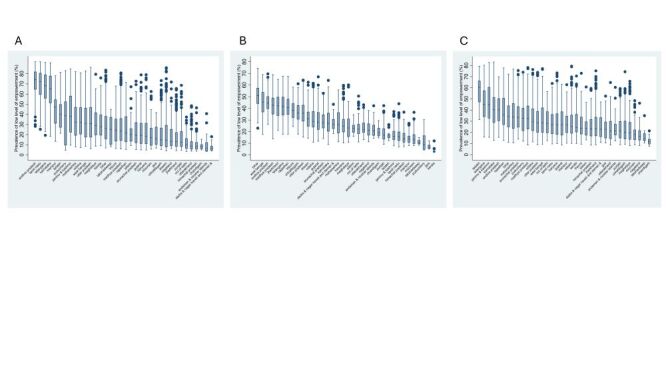
Within-state community variation in women’s low level of empowerment in India. **Panel A**. Attitude to violence. **Panel B**. Social independence. **Panel C**. Decision-making. A total of 9104 communities, 720 districts, and 36 states are included.

Box plots illustrating within-state community variation in women’s low level of empowerment revealed a substantially large variation across all three domains (**Figure 2**). In the attitude to violence domain, for example, Andhra Pradesh had the lowest performance in women’s empowerment, with community prevalence of low level of empowerment ranging from 28.2 to 91.8%. Even the best-performing state, Dadra & Nagar Haveli and Daman & Diu, showed notable community variation, ranging from 3.9 to 18.0%. The decision-making domain exhibited the most pronounced within-state community variation, with most states showing high disparities. Karnataka, for example, demonstrated the widest range, with community prevalence of low level of empowerment spanning from 12.8 to 83.1%.

### Within-district community variation in women’s low level of empowerment

Overall, Pearson correlation showed a moderate to strong correlation between district mean prevalence and within-district community variation (SD for each district) across three domains (Pearson correlation (*r*) = 0.41 ~ 0.72, *P* < 0.001) (Figure S2 in the **Online Supplementary Document**). That is, districts that performed poorly in women’s empowerment were often characterised by substantial inequalities in women’s empowerment between their communities. Nonetheless, multiple districts with above-average performance also exhibited substantial disparities between communities. For example, in the attitude to violence domain where the weakest linear relationship was found, 171 districts had a prevalence of women’s low level of empowerment below the all-India mean and a within-district SD above its mean, followed by 108 districts in the decision-making domain and 81 districts in the social independence domain.

### Geographic distribution of under-empowered communities

**Figure 3:**
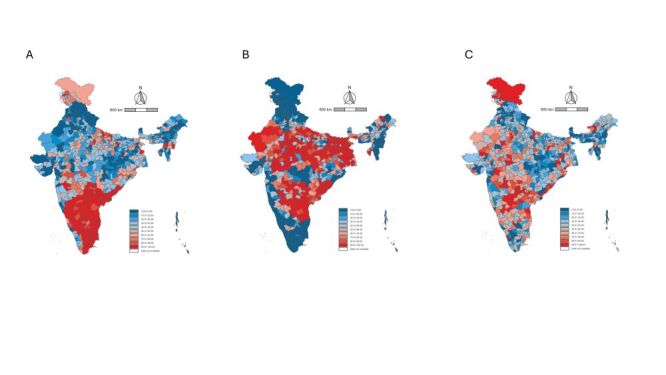
Geographic distribution of under-empowered communities across 720 districts in India. **Panel A.** Attitude to violence. **Panel B.** Social independence. **Panel C.** Decision-making. A total of 9104 communities, 720 districts, and 36 states are included. Under-empowered communities are defined as those with a prevalence of low level of empowerment equal to or higher than the national mean of low level of empowerment.

The spatial distribution of under-empowered communities across 720 districts revealed distinct patterns depending on the domain of women’s empowerment (**Figure 3**; Table S3–4 in the **Online Supplementary Document**). In the attitude to violence domain, under-empowered communities were heavily concentrated in the southern region of India. Out of 720 districts, 119 districts had more than 90% under-empowered communities in this domain, with 106 districts of these located in the south (*e.g*. Guntur, Srikakulam, Jangoan, Medak, Chennai, and Dharmapuri). Meanwhile, in the social independence domain, under-empowered communities were mainly concentrated in the northern, central, and eastern regions. Among the 210 districts with more than 90% under-empowered communities in this domain, 151 districts were located in these regions, including 78 districts from the north (*e.g*. Pashchim Champaran, Gaya, Etah, Fatehpur, and Churu). At the same time, some parts of the southern region performed poorly in both the attitude to violence and the social independence domain, with 36 districts having more than 90% under-empowered communities in both domains (*e.g*. Srikakulam and Jangoan). In the decision-making domain, under-empowered communities were more widely dispersed, with no significant concentration in specific districts or regions.

## DISCUSSION

Using a globally validated and comprehensive measure of women’s empowerment – the SWPER, this study quantified the geographic variation in women’s empowerment across the key geographic levels in India: state, district, and community. To the best of our knowledge, this is the first study to examine regional inequalities in women’s empowerment at multiple geographic levels, extending beyond previous research that primarily focused on state-level geographic variations of women’s empowerment [8,11,31].

India, a nation of immense diversity, stands out for its vast population and rich tapestry of local variations in governance, socioeconomic dynamics, and cultural traditions. This heterogeneity brings both opportunities and challenges, emphasising the need for precise geographic targeting in policy design. Developing effective women’s empowerment strategies begins with identifying the most relevant administrative or regional units to ensure efficient use of limited resources. Our multilevel analysis revealed that the largest variation in women’s empowerment occurred at the community level (except for the social independence domain), whereas districts were the least significant contributor. Large community variation was observed within states, with districts that demonstrated above-average performance in empowerment still showing considerable inequalities between communities.

Urban-rural distinctions, local governance, and community-specific cultural traditions may result in localised disparities in women’s status, even within broader regions that show progress. Rural communities often experience limited infrastructure, shortages of trained health professionals, and inadequate service provision [46]. In contrast, urban areas generally provide greater economic opportunities and institutional support, yet intra-district disparities persist, particularly in peri-urban settlements where infrastructure and social services remain insufficient [47]. Additionally, local labour market structures within districts may shape women’s economic agency at the community level, as employment opportunities vary based on local government initiatives and economic conditions. While some communities benefit from targeted training and skill development programmes, others rely on restrictive labour conditions that limit women’s workforce participation [48]. Caste-based communities further illustrate how women face dual burden of caste hierarchy and gender discrimination, as exemplified by the historical marginalisation and exploitation of Dalit women at the intersections of caste, class, and gender [49]. These variations highlight that structural improvements at the district or state level do not necessarily translate into uniform progress across all communities, and relying solely on state or district averages risks overlooking under-empowered communities nested within these regions. Notably, the Mahila Samakhya (Women’s Empowerment through Awareness and Education) in India is one of the examples of community-based initiatives addressing gender disparity in education, particularly working through marginalised women’s collectives known as sanghas, and local government officials known as panchayats, to address the specific needs of rural women [50].

At the same time, we found significant differences in geographic patterns across each domain of women’s empowerment. In the attitude to violence domain, for instance, the largest variation was observed at the community level (47.1%), though state-level variation was comparably high (41.7%). We also found that under-empowered communities in this domain were predominantly concentrated in southern India. Conversely, in the social independence domain, states accounted for the largest share of variation (57.7%), with under-empowered communities under this domain clustering mainly in northern and central regions. Lastly, the decision-making domain exhibited a substantial community-level variation (69.6%), with under-empowered communities dispersed across India rather than concentrated in specific districts or states.

The significant community-level variations in the attitude to violence domain highlight the entrenched nature of localised gender norms regarding gender-based violence. In many Indian communities, traditional beliefs and patriarchal structures perpetuate the notion that men have authority over women, leading to the normalisation of intimate partner violence (IPV). An ethnographic study by Ghosh (2011) highlights how community norms legitimise wife-beating as a form of discipline or punishment when women are perceived to disrespect their in-laws or neglect household chores [51]. The study further explains that community pressures force women to condone the idea of IPV, particularly through the influential role of khap panchayats (caste-based assemblies composed of local elders who hold authority over community affairs). This form of misogyny against women is often manifested through the boycott of one’s own community members, regardless of the agreements or legislations from larger districts or states [51]. Meanwhile, the comparably high state-level variation in this domain may reflect the pronounced justification of wife-beating observed in certain southern states. Given the positive association between women’s justification of wife-beating and women’s actual IPV experience [52], this finding aligns with prior studies showing higher odds of men’s IPV perpetration in southern India compared to the north [53,54], which requires more in-depth analysis for this regional variation.

In the domain of social independence, which includes women’s education attainment, age at marriage, and media exposure, the state was the predominant factor influencing the variation. This pattern underscores the critical influence of state-level policies and programmes in shaping women’s empowerment within this domain. A key example is the Right to Education (RTE) Act started in 2009, which mandates free and compulsory education for children aged six to 14 years [55]. While the RTE Act is a national policy, its implementation and effectiveness vary widely across states. Southern states, including Kerala and Tamil Nadu, have leveraged the RTE Act alongside their respective education initiatives, such as Kerala’s Infrastructure and Technology for Education initiative [56] and Tamil Nadu’s mid-day meal schemes [57], to enhance the learning environment and reduce dropout rates among girls. These efforts have led to better educational outcomes and a higher average age of marriage, as exemplified by Kerala, known for its highest female literacy rate [8] and relatively low prevalence of child marriage [58]. In contrast, northern states including Bihar and Uttar Pradesh, where under-empowered communities in this domain were mainly clustered, are widely recognised as under-developed states with poor economic conditions and infrastructure deficiencies [8,31]. Bihar, in particular, struggles with low-quality education systems, marked by a shortage of qualified teachers [59] and low literacy rates [8]. These deficiencies may be closely linked to the state’s high prevalence of child marriage [60], further hindering progress in women’s social independence.

The overwhelming community-level variation in the decision-making domain reflects the influence of localised household dynamics. Notably, the wide dispersion of under-empowered communities in this domain highlights the role of spousal (interpersonal) relationships and family arrangements (*e.g*. co-residence with parents-in-law) in shaping intra-household decision-making processes. First, studies highlight that in a supportive marital relationship, women equally participate in household decision-making with their husbands, particularly in matters related to finances, health care, and mobility [61]. Second, in households where women live with their in-laws, especially in traditional joint family structures, decision-making authority often resides with senior family members, particularly in communities where societal norms reinforce male and elder dominance [62]. In contrast, nuclear family arrangements or matrilineal communities provide women more control over household decisions, as these structures are more conducive to female autonomy. Women of the matrilineal Khai tribe of Meghalaya, for example, have been major property custodians and played a central role in household decisions, notwithstanding recent criticism that their engagement in public and political spheres remains limited [63].

Further examination is warranted regarding the clustering of under-empowered communities in the attitude to violence domain in southern India, a region generally known for its better socioeconomic development but also in terms of overall women’s status compared to the northern states [8]. The relatively high socioeconomic status of women in southern states is evident in the geographic pattern of under-empowered communities in the social independence domain, where such communities are notably absent in this region. In particular, we focus on Tamil Nadu, one of the southern states previously deemed as a state where women’s empowerment level is very high [31]. The government of Tamil Nadu launched various policies to improve women’s empowerment including Periyar EVR Nagammai Scheme, which provides free education to female students irrespective of caste, creed, and community [64]. Also, self-help groups to promote rural women’s socioeconomic empowerment have been formed and monitored through Tamil Nadu Corporations for Development of Women, resulting in roughly 92% of women having savings or bank accounts [31].

Therefore, our finding of unexpectedly high justification of wife-beating observed in the southern region is quite striking, as women’s elevated socioeconomic status did not necessarily translate into their lower acceptance of violence. Rather, our findings suggest that women’s higher socioeconomic status alone is not sufficient to break the patriarchal beliefs. In fact, recent studies increasingly note that women’s increased economic power and agency can sometimes threaten men’s control or power in the relationship, exacerbating women’s vulnerabilities to IPV [8,65]. Additionally, in India where divorce is rare and socially stigmatised, even financially independent women may feel pressured to remain in abusive relationships [65]. This co-occurrence of women’s higher socioeconomic status and risk of violence is not unique to India. A study across 27 European countries found that disparities in educational attainment between partners were associated with a higher IPV risk, particularly when women’s education exceeded that of their husbands [66]. Similarly, a global study of 44 countries found that in societies where few women participate in paid labour, women who work for cash face a greater risk of IPV [67]. Considering the limitations of government education schemes which are often strongly embedded within social norms and practices [68], efforts to empower women should extend beyond increasing the years of schooling to actively challenging harmful gender norms that normalise domestic violence.

Collectively, the differing geographic variations across domains of women’s empowerment highlight the importance of examining each domain separately while also addressing them comprehensively, aligning with prior research that emphasises empowerment as a multidimensional construct rather than reducing it to a single composite index [32,69]. Expanding educational systems or employment opportunities is undoubtedly vital for advancing women’s social independence; however, it alone cannot dismantle entrenched gender norms or gender power relationships within the household. Tackling these challenges requires an integrated approach that simultaneously targets multiple aspects of empowerment. One notable example is Self-Help Group-Based Intervention for Combating Violence Against Women (SHGIVAW) in Rajasthan, a community-led initiative which mobilises women as economic actors but also as challengers against gender-based violence, by combining a microfinance programme with participatory training on domestic violence, gender norms, and sexuality [70]. As programmes like SHGIVAW illustrate, women’s empowerment is not monolithic; given its multifaceted nature, policymakers and intervention developers should design layered strategies in which economic, educational, and normative shifts are pursued in an integrated manner to ultimately foster holistic and sustainable empowerment for women.

Our study has a few limitations. The SWPER index, while comprehensive, does not entirely capture the multidimensional nature of women’s empowerment. Notably, women’s economic participation is omitted in the global version of SWPER due to concerns that women’s labour participation might stem from necessity rather than choice [32]. Nevertheless, SWPER remains one of the comprehensive and validated tools for measuring women’s empowerment in the context of low- and middle-income countries. Also, the items in SWPER rely on women’s self-reports, which may be subject to errors influenced by social desirability or recall bias. Additionally, since SWPER is designed for currently married women, this study does not capture the empowerment experiences of unmarried adolescents or divorced/widowed women, whose empowerment trajectories and gendered challenges may differ from those of women in union. There is also the possibility of selection bias due to missing data. A total of 227 women were excluded due to missing SWPER responses, but multiple imputation analyses confirmed that VPC remained consistent (data not shown), suggesting minimal impact on the results. Furthermore, the primary sampling units or clusters in NFHS may not accurately reflect the community concept. Even so, their clear geographic boundaries and representation of local populations have provided a practical and widely utilised approximation of communities in multilevel studies [71]. Going further, we were unable to identify which specific communities experience the highest burden of women’s disempowerment. Future research should build on this by employing advanced mapping techniques, such as integrating Census and DHS data, to pinpoint these disparities more precisely [26]. Field studies and qualitative research may enhance the accuracy and contextual understanding of geographic patterns and provide more targeted insights for effective policy interventions. Finally, this study could not assess the extent to which contextual factors, such as state-level employment programmes or gender-inequitable legislation, explain geographic disparities in women’s empowerment due to data limitations. Future research should integrate such contextual variables to identify the structural barriers at different geographic levels, providing clearer insights for targeted policy interventions.

## CONCLUSIONS

This study underscores the critical need for precise geographic targeting and a multifaceted approach to advancing women’s empowerment in India, a nation marked by immense diversity and local variations. Our findings reveal that community-level variation is most pronounced in domains such as attitude to violence and decision-making, highlighting the need for localised, community-targeted interventions. Conversely, the significant state-level variation in social independence domain emphasises the continued relevance of state-level policy design. Furthermore, the persistence of conservative gendered attitudes in regions with higher educational attainment points to the need for integrated strategies that address all domains of empowerment collectively. Also, the emphasis within this framework may vary by region – for instance, shifting norms around violence acceptance may be more critical in southern India, while generally expanding access to formal education itself may be a greater priority in the north. Ensuring that women’s empowerment strategies balance these regional needs within a comprehensive, multilevel approach will be essential for achieving meaningful and sustainable progress in women’s empowerment in India.

## Additional material


Online Supplementary Document
