# Supplementary material for: Geographic variation in women’s empowerment: a multilevel analysis of India’s National Family Health Survey 2021
Source: J Glob Health. 2025 Jun 27;15:04159. doi: 10.7189/jogh.15.04159 (PMC12203628; doi:10.7189/jogh.15.04159)
Supplement: Online Supplementary Document [file jogh-15-04159-s001.pdf]

Figure S1. Flow diagram showing exclusions and final sample sizes for the study population.

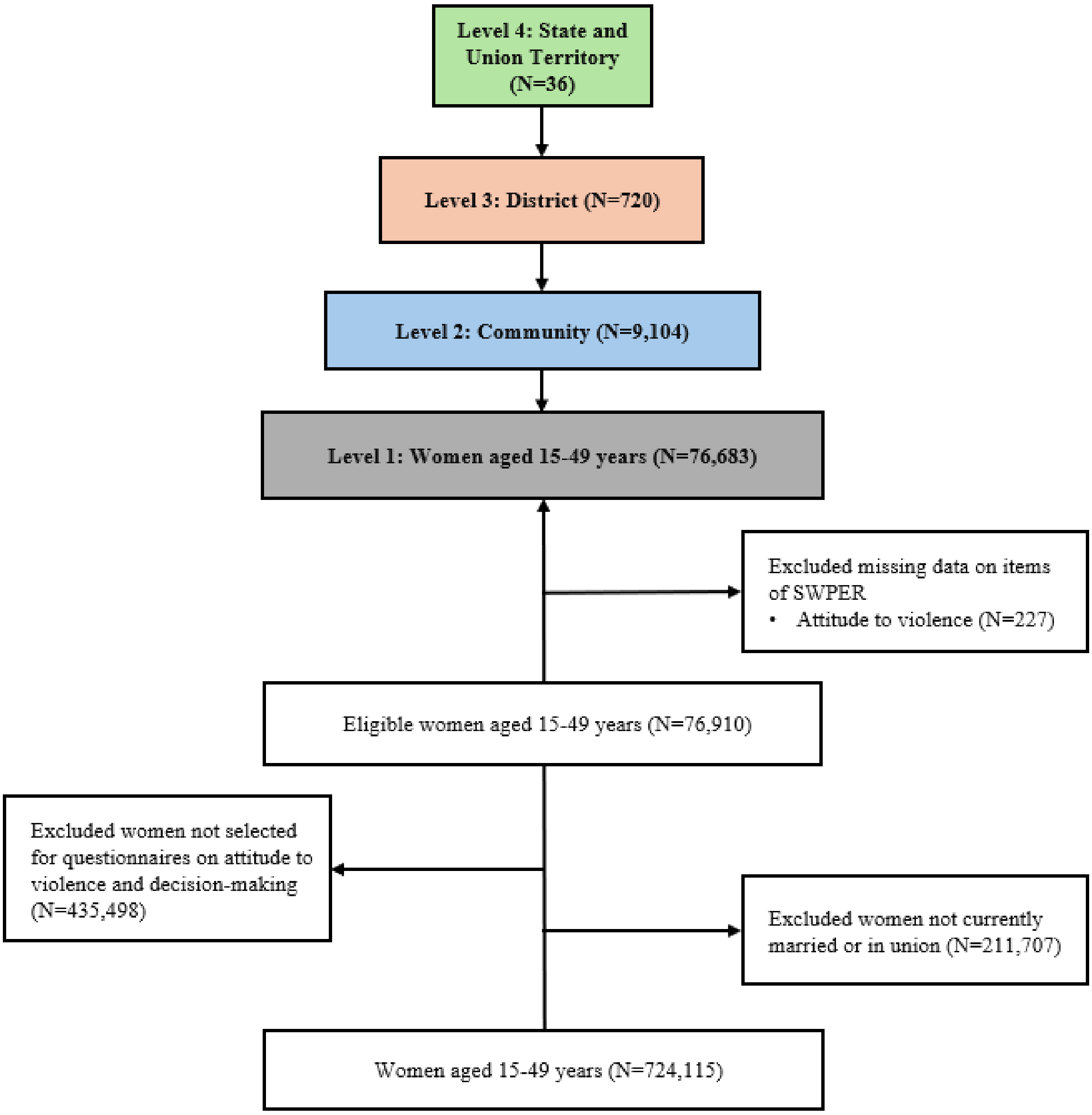

Table S1. Coding structure for each of the 14 items used for SWPER index.

Domain	Variable	Coding
**Attitude to violence**	**Beating justified if:**	Yes = -1;<br>Don't Know = 0;<br>No = 1
	1. Wife goes out without telling husband	
	2. Wife neglects the children	
	3. Wife argues with husband	
	4. Wife refuses to have sex with husband	
	5. Wife burns the food	
**Social independence**	6. Frequency of reading newspaper or magazine	Not at all = 0<br>Less than once a week = 1<br>At least once a week = 2
	7. Woman's education in completed years of schooling	Years
	8. Education difference: woman's minus husband's years of schooling	Years
	9. Age difference: woman's minus husband's age	Years
	10. Age of woman at first cohabitation	Years
	11. Age of woman first birth	Years
**Decision-making**	**Who usually decides on:**	Husband/other alone = -1;<br>Joint or respondent alone = 1
	12. Women's health care	
	13. Large household purchases	
	14. Visits to family or relatives	

Retrieved from Ewerling F, Raj A, Victora CG, Hellwig F, Coll CV, Barros AJ. SWPER Global: A survey-based women's empowerment index expanded from Africa to all low-and middle-income countries. Journal of global health 2020; 10(2).

Table S2. Distribution of number of districts, communities, and women by 36 states or Union Territories.

	State or Union Territory	No. of districts	No. of communities	No. of women
1	Andaman & Nicobar islands	3	38	264
2	Andhra Pradesh	26	164	1,242
3	Arunachal Pradesh	20	259	2,046
4	Assam	33	419	3,912
5	Bihar	38	517	4,643
6	Chandigarh	1	12	83
7	Chhattisgarh	27	343	2,817
8	Dadra & Nagar Haveli and Daman & Diu	3	39	291
9	Goa	2	26	190
10	Gujarat	33	419	3,709
11	Haryana	22	262	2,347
12	Himachal Pradesh	12	154	1,219
13	Jammu & Kashmir	20	253	2,014
14	Jharkhand	24	328	2,906
15	Karnataka	30	380	3,235
16	Kerala	14	180	1,233
17	Ladakh	2	25	187
18	Lakshadweep	1	13	120
19	Madhya Pradesh	51	619	5,073
20	Maharashtra	36	454	3,753
21	Manipur	9	117	816
22	Meghalaya	11	140	1,252
23	Mizoram	8	102	651
24	Nagaland	11	141	879
25	Nct of Delhi	11	138	1,100
26	Odisha	30	386	3,098
27	Puducherry	4	51	388
28	Punjab	22	280	2,352
29	Rajasthan	33	451	4,722
30	Sikkim	4	50	318
31	Tamil Nadu	32	407	2,739
32	Telangana	31	397	3,080
33	Tripura	8	100	906
34	Uttar Pradesh	75	1,012	9,324
35	Uttarakhand	13	175	1,311
36	West Bengal	20	253	2,463
	**Total**	**720**	**9,104**	**76,683**

Figure S2. Correlation between district mean prevalence and within-district community variation in women's low level of empowerment in India.

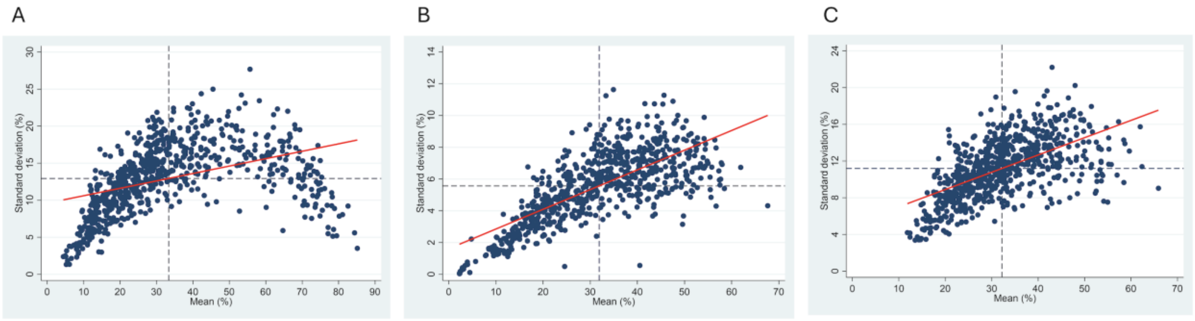

Note.

- Panel A. Attitude to violence. Panel B. Social independence. Panel C. Decision-making.
- Attitude to violence: Pearson's $r = 0.41$ ($P < 0.001$); social independence: Pearson's $r = 0.72$ ($P < 0.001$); decision-making: Pearson's $r = 0.58$ ($P < 0.001$).
- Out of a total of 720 districts, Alluri Sitharama Raju in Andhra Pradesh is excluded since it had only one community in the dataset.

Table S3. Number of districts in each category in terms of geographic distribution of under-empowered communities.

	Category (%)	Number of districts		
		**Attitude to violence**	**Social independence**	**Decision-making**
1	0.00-10.00	175	239	106
2	10.01-20.00	66	26	70
3	20.01-30.00	72	30	73
4	30.01-40.00	84	41	93
5	40.01-50.00	69	28	106
6	50.01-60.00	37	32	51
7	60.01-70.00	42	43	83
8	70.01-80.00	30	31	47
9	80.01-90.00	26	40	32
10	90.01-100.00	119	210	59
	**Total**	**720**	**720**	**720**

Table S4. Proportion (%) of under-empowered communities across 720 districts.

State	District	District ID	Proportion (%) of under-empowered communities.		
			Attitude to violence	Social independence	Decision-making
Andhra pradesh	Alluri Sitharama Raju	2	100.00	100.00	0.00
Andhra pradesh	Anakapalli	3	100.00	100.00	100.00
Andhra pradesh	Ananthapuramu	4	100.00	100.00	57.14
Andhra pradesh	Annamayya	5	100.00	100.00	62.50
Andhra pradesh	Bapatla	6	100.00	100.00	60.00
Andhra pradesh	Chittoor	7	100.00	100.00	50.00
Andhra pradesh	Dr. B.R. Ambedkar Konaseema	8	100.00	100.00	0.00
Andhra pradesh	East Godavari	9	100.00	100.00	71.43
Andhra pradesh	Eluru	10	100.00	83.33	66.67
Andhra pradesh	Guntur	11	100.00	87.50	87.50
Andhra pradesh	Kakinada	12	100.00	83.33	100.00
Andhra pradesh	Krishna	13	100.00	80.00	80.00
Andhra pradesh	Kurnool	14	100.00	100.00	83.33
Andhra pradesh	Nandyal	15	100.00	100.00	14.29
Andhra pradesh	Ntr	16	100.00	100.00	83.33
Andhra pradesh	Palnadu	17	100.00	100.00	75.00
Andhra pradesh	Parvathipuram Manyam	18	100.00	100.00	100.00
Andhra pradesh	Prakasam	19	85.71	100.00	28.57
Andhra pradesh	Sri Potti Sriramulu Nellore	20	90.91	100.00	100.00
Andhra pradesh	Sri Sathya Sai	21	100.00	100.00	50.00
Andhra pradesh	Srikakulam	22	100.00	100.00	90.91
Andhra pradesh	Tirupati	23	100.00	100.00	83.33
Andhra pradesh	Visakhapatnam	24	100.00	100.00	85.71
Andhra pradesh	Vizianagaram	25	100.00	100.00	81.82
Andhra pradesh	West Godavari	26	100.00	75.00	50.00
Andhra pradesh	Y.S.R.	27	100.00	88.89	77.78
West bengal	Kolkata	29	30.77	15.38	0.00
Jammu & kashmir	Kupwara	30	38.46	0.00	46.15
Jammu & kashmir	Badgam	31	46.15	0.00	100.00
Ladakh	Leh	32	66.67	0.00	100.00
Jammu & kashmir	Punch	33	61.54	7.69	92.31
Jammu & kashmir	Rajouri	34	69.23	0.00	38.46
Jammu & kashmir	Kathua	35	30.77	0.00	92.31
Jammu & kashmir	Baramula	36	91.67	0.00	83.33
Jammu & kashmir	Bandipore	37	84.62	0.00	100.00
Jammu & kashmir	Srinagar	38	33.33	0.00	75.00
Jammu & kashmir	Ganderbal	39	84.62	30.77	84.62
Jammu & kashmir	Pulwama	40	23.08	0.00	76.92
Jammu & kashmir	Shupiyan	41	38.46	0.00	61.54
Jammu & kashmir	Anantnag	42	66.67	0.00	100.00
Jammu & kashmir	Kulgam	43	58.33	0.00	91.67
Jammu & kashmir	Doda	44	76.92	0.00	46.15
Jammu & kashmir	Ramban	45	53.85	0.00	100.00
Jammu & kashmir	Kishtwar	46	76.92	0.00	92.31
Jammu & kashmir	Udhampur	47	58.33	0.00	41.67
Jammu & kashmir	Reasi	48	66.67	8.33	50.00
Jammu & kashmir	Jammu	49	75.00	0.00	75.00
Jammu & kashmir	Samba	50	38.46	0.00	69.23
Himachal pradesh	Chamba	51	0.00	0.00	0.00
Himachal pradesh	Kangra	52	0.00	0.00	38.46
Himachal pradesh	Lahul & Spiti	53	0.00	0.00	23.08
Himachal pradesh	Kullu	54	0.00	8.33	33.33
Himachal pradesh	Mandi	55	0.00	0.00	23.08
Himachal pradesh	Hamirpur	56	0.00	0.00	30.77
Himachal pradesh	Una	57	0.00	0.00	41.67
Himachal pradesh	Bilaspur	58	0.00	0.00	38.46
Himachal pradesh	Solan	59	7.69	0.00	15.38

Himachal pradesh	Sirmaur	60	0.00	0.00	7.69
Himachal pradesh	Shimla	61	0.00	0.00	7.69
Himachal pradesh	Kinnaur	62	7.69	0.00	38.46
Punjab	Kapurthala	63	23.08	0.00	38.46
Punjab	Jalandhar	64	0.00	0.00	7.69
Punjab	Hoshiarpur	65	7.69	0.00	7.69
Punjab	Shahid Bhagat Singh Nagar	66	7.69	0.00	0.00
Punjab	Fatehgarh Sahib	67	7.69	0.00	0.00
Punjab	Ludhiana	68	0.00	0.00	15.38
Punjab	Moga	69	7.69	0.00	30.77
Punjab	Muktsar	70	16.67	33.33	33.33
Punjab	Faridkot	71	8.33	0.00	50.00
Punjab	Bathinda	72	84.62	15.38	100.00
Punjab	Mansa	73	23.08	7.69	30.77
Punjab	Patiala	74	0.00	0.00	23.08
Punjab	Amritsar	75	7.69	0.00	7.69
Punjab	Tarn Taran	76	0.00	7.69	30.77
Punjab	Rupnagar	77	0.00	0.00	18.18
Punjab	Sahibzada Ajit Singh Nagar	78	0.00	0.00	15.38
Punjab	Sangrur	79	15.38	0.00	7.69
Punjab	Barnala	80	15.38	0.00	0.00
Chandigarh	Chandigarh	81	0.00	0.00	0.00
Uttarakhand	Uttarkashi	82	0.00	7.14	28.57
Uttarakhand	Chamoli	83	0.00	0.00	7.69
Uttarakhand	Rudraprayag	84	0.00	7.14	21.43
Uttarakhand	Tehri Garhwal	85	0.00	35.71	28.57
Uttarakhand	Dehradun	86	16.67	16.67	50.00
Uttarakhand	Garhwal	87	7.69	0.00	15.38
Uttarakhand	Pithoragarh	88	0.00	7.14	7.14
Uttarakhand	Bageshwar	89	0.00	0.00	7.14
Uttarakhand	Almora	90	7.14	0.00	28.57
Uttarakhand	Champawat	91	0.00	30.77	0.00
Uttarakhand	Nainital	92	0.00	15.38	46.15
Uttarakhand	Udham Singh Nagar	93	7.14	50.00	7.14
Uttarakhand	Hardwar	94	46.15	0.00	46.15
Haryana	Panchkula	95	0.00	0.00	9.09
Haryana	Ambala	96	38.46	0.00	61.54
Haryana	Yamunanagar	97	38.46	0.00	53.85
Haryana	Kurukshetra	98	25.00	8.33	33.33
Haryana	Kaithal	99	0.00	33.33	33.33
Haryana	Karnal	100	23.08	7.69	46.15
Haryana	Panipat	101	46.15	7.69	53.85
Haryana	Sonipat	102	15.38	0.00	23.08
Haryana	Jind	103	23.08	69.23	38.46
Haryana	Fatehabad	104	8.33	16.67	16.67
Haryana	Sirsa	105	33.33	0.00	0.00
Haryana	Hisar	106	0.00	23.08	7.69
Haryana	Rohtak	107	0.00	0.00	25.00
Haryana	Jhajjar	108	8.33	16.67	41.67
Haryana	Mahendragarh	109	30.77	7.69	38.46
Haryana	Rewari	110	54.55	0.00	45.45
Haryana	Gurgaon	111	0.00	0.00	8.33
Haryana	Mewat	112	69.23	100.00	76.92
Haryana	Faridabad	113	30.77	38.46	30.77
Haryana	Palwal	114	23.08	92.31	76.92
Rajasthan	Ganganagar	115	0.00	71.43	28.57
Rajasthan	Hanumangarh	116	0.00	71.43	50.00
Rajasthan	Bikaner	117	7.14	92.86	64.29
Rajasthan	Churu	118	28.57	92.86	64.29
Rajasthan	Jhunjhunun	119	0.00	85.71	35.71
Rajasthan	Alwar	120	15.38	92.31	61.54
Rajasthan	Bharatpur	121	50.00	85.71	100.00
Rajasthan	Dhaulpur	122	57.14	100.00	64.29
Rajasthan	Karauli	123	46.15	100.00	84.62

Rajasthan	Sawai Madhopur	124	71.43	100.00	78.57
Rajasthan	Dausa	125	42.86	100.00	78.57
Rajasthan	Jaipur	126	15.38	30.77	30.77
Rajasthan	Sikar	127	30.77	100.00	69.23
Rajasthan	Nagaur	128	28.57	85.71	71.43
Rajasthan	Jodhpur	129	14.29	85.71	50.00
Rajasthan	Jaisalmer	130	28.57	100.00	64.29
Rajasthan	Barmer	131	21.43	92.86	57.14
Rajasthan	Jalor	132	38.46	92.31	84.62
Rajasthan	Sirohi	133	50.00	57.14	71.43
Rajasthan	Pali	134	0.00	57.14	78.57
Rajasthan	Ajmer	135	15.38	61.54	69.23
Rajasthan	Tonk	136	57.14	100.00	92.86
Rajasthan	Bundi	137	50.00	92.86	71.43
Rajasthan	Bhilwara	138	28.57	85.71	7.14
Rajasthan	Rajsamand	139	7.69	76.92	30.77
Rajasthan	Dungarpur	140	14.29	50.00	57.14
Rajasthan	Banswara	141	21.43	92.86	64.29
Rajasthan	Chittaurgarh	142	30.77	92.31	69.23
Rajasthan	Kota	143	38.46	23.08	61.54
Rajasthan	Baran	144	57.14	92.86	64.29
Rajasthan	Jhalawar	145	38.46	92.31	53.85
Rajasthan	Udaipur	146	50.00	85.71	57.14
Rajasthan	Pratapgarh	147	76.92	69.23	38.46
Uttar pradesh	Saharanpur	148	7.69	46.15	7.69
Uttar pradesh	Bijnor	149	30.77	46.15	46.15
Uttar pradesh	Rampur	150	27.27	72.73	63.64
Uttar pradesh	Jyotiba Phule Nagar	151	7.69	53.85	53.85
Uttar pradesh	Meerut	152	35.71	57.14	35.71
Uttar pradesh	Baghpat	153	14.29	50.00	28.57
Uttar pradesh	Gautam Buddha Nagar	154	18.18	36.36	45.45
Uttar pradesh	Bulandshahr	155	7.69	69.23	15.38
Uttar pradesh	Aligarh	156	15.38	69.23	15.38
Uttar pradesh	Mahamaya Nagar	157	21.43	78.57	92.86
Uttar pradesh	Mathura	158	42.86	85.71	71.43
Uttar pradesh	Agra	159	41.67	100.00	50.00
Uttar pradesh	Firozabad	160	21.43	64.29	57.14
Uttar pradesh	Mainpuri	161	7.69	30.77	23.08
Uttar pradesh	Bareilly	162	21.43	100.00	57.14
Uttar pradesh	Pilibhit	163	15.38	76.92	15.38
Uttar pradesh	Shahjahanpur	164	14.29	92.86	35.71
Uttar pradesh	Sitapur	165	42.86	85.71	28.57
Uttar pradesh	Hardoi	166	71.43	92.86	42.86
Uttar pradesh	Unnao	167	61.54	61.54	46.15
Uttar pradesh	Lucknow	168	42.86	21.43	0.00
Uttar pradesh	Farrukhabad	169	57.14	85.71	42.86
Uttar pradesh	Kannauj	170	46.15	53.85	38.46
Uttar pradesh	Etawah	171	42.86	35.71	50.00
Uttar pradesh	Auraiya	172	38.46	7.69	15.38
Uttar pradesh	Kanpur Dehat	173	92.31	46.15	46.15
Uttar pradesh	Kanpur Nagar	174	57.14	21.43	57.14
Uttar pradesh	Jalaun	175	84.62	100.00	46.15
Uttar pradesh	Jhansi	176	7.14	64.29	14.29
Uttar pradesh	Lalitpur	177	71.43	100.00	42.86
Uttar pradesh	Hamirpur	178	42.86	78.57	21.43
Uttar pradesh	Mahoba	179	42.86	100.00	21.43
Uttar pradesh	Banda	180	53.85	92.31	69.23
Uttar pradesh	Chitrakoot	181	61.54	92.31	46.15
Uttar pradesh	Fatehpur	182	35.71	92.86	50.00
Uttar pradesh	Pratapgarh	183	35.71	28.57	14.29
Uttar pradesh	Kaushambi	184	46.15	84.62	15.38
Uttar pradesh	Allahabad	185	66.67	91.67	41.67
Uttar pradesh	Bara Banki	186	85.71	92.86	100.00
Uttar pradesh	Faizabad	187	35.71	0.00	42.86
Uttar pradesh	Ambedkar Nagar	188	85.71	57.14	21.43

Uttar pradesh	Bahraich	189	100.00	92.31	100.00
Uttar pradesh	Shrawasti	190	78.57	100.00	64.29
Chhattisgarh	Balrampur	191	15.38	100.00	15.38
Uttar pradesh	Gonda	192	78.57	78.57	50.00
Uttar pradesh	Siddharthnagar	193	92.86	85.71	64.29
Uttar pradesh	Basti	194	42.86	64.29	21.43
Uttar pradesh	Sant Kabir Nagar	195	92.86	100.00	14.29
Uttar pradesh	Maharajganj	196	50.00	64.29	28.57
Uttar pradesh	Gorakhpur	197	38.46	61.54	23.08
Uttar pradesh	Kushinagar	198	50.00	42.86	14.29
Uttar pradesh	Deoria	199	28.57	28.57	57.14
Uttar pradesh	Azamgarh	200	46.15	15.38	15.38
Uttar pradesh	Mau	201	64.29	7.14	78.57
Uttar pradesh	Ballia	202	100.00	0.00	30.77
Uttar pradesh	Jaunpur	203	28.57	50.00	57.14
Uttar pradesh	Ghazipur	204	78.57	42.86	21.43
Uttar pradesh	Chandauli	205	42.86	28.57	14.29
Uttar pradesh	Varanasi	206	78.57	0.00	50.00
Uttar pradesh	Sant Ravidas Nagar	207	61.54	100.00	46.15
Uttar pradesh	Mirzapur	208	50.00	57.14	35.71
Uttar pradesh	Sonbhadra	209	76.92	100.00	46.15
Uttar pradesh	Etah	210	38.46	100.00	46.15
Uttar pradesh	Kanshiram Nagar	211	21.43	85.71	78.57
Bihar	Pashchim Champaran	212	30.77	100.00	30.77
Bihar	Purba Champaran	213	15.38	100.00	30.77
Bihar	Sheohar	214	28.57	100.00	57.14
Bihar	Sitamarhi	215	7.14	92.86	28.57
Bihar	Madhubani	216	28.57	100.00	28.57
Bihar	Supaul	217	14.29	100.00	0.00
Bihar	Araria	218	64.29	100.00	28.57
Bihar	Kishanganj	219	69.23	100.00	84.62
Bihar	Purnia	220	42.86	100.00	78.57
Bihar	Katihar	221	0.00	100.00	84.62
Bihar	Madhepura	222	21.43	100.00	21.43
Bihar	Saharsa	223	38.46	100.00	53.85
Bihar	Darbhanga	224	30.77	100.00	46.15
Bihar	Muzaffarpur	225	38.46	100.00	38.46
Bihar	Gopalganj	226	0.00	100.00	50.00
Bihar	Siwan	227	57.14	100.00	14.29
Bihar	Saran	228	30.77	100.00	69.23
Bihar	Vaishali	229	42.86	100.00	50.00
Bihar	Samastipur	230	28.57	100.00	42.86
Bihar	Begusarai	231	35.71	100.00	57.14
Bihar	Khagaria	232	50.00	100.00	50.00
Bihar	Bhagalpur	233	71.43	92.86	71.43
Bihar	Banka	234	42.86	100.00	57.14
Bihar	Munger	235	42.86	57.14	71.43
Bihar	Lakhisarai	236	71.43	100.00	42.86
Bihar	Sheikhpura	237	23.08	100.00	61.54
Bihar	Nalanda	238	38.46	100.00	46.15
Bihar	Patna	239	28.57	85.71	85.71
Bihar	Bhojpur	240	42.86	100.00	78.57
Bihar	Buxer	241	7.69	92.31	69.23
Bihar	Kaimur (Bhabua)	242	0.00	92.86	71.43
Bihar	Rohtas	243	15.38	92.31	92.31
Bihar	Gaya	244	14.29	100.00	35.71
Bihar	Nawada	245	46.15	100.00	61.54
Bihar	Jamui	246	69.23	100.00	69.23
Bihar	Jehanabad	247	42.86	100.00	85.71
Bihar	Arwal	248	28.57	100.00	92.86
Sikkim	North District	249	8.33	25.00	0.00
Sikkim	West District	250	23.08	0.00	30.77
Sikkim	South District	251	23.08	0.00	15.38
Sikkim	East District	252	0.00	0.00	25.00
Arunachal pradesh	Tawang	253	0.00	7.14	92.86

Arunachal pradesh	West Kameng	254	21.43	0.00	78.57
Arunachal pradesh	East Kameng	255	61.54	30.77	92.31
Arunachal pradesh	Papum Pare	256	57.14	57.14	42.86
Arunachal pradesh	Upper Subansiri	257	30.77	61.54	38.46
Arunachal pradesh	Upper Siang	258	15.38	92.31	30.77
Arunachal pradesh	Changlang	259	0.00	42.86	28.57
Arunachal pradesh	Lower Subansiri	260	38.46	84.62	61.54
Arunachal pradesh	Dibang Valley	261	28.57	0.00	50.00
Arunachal pradesh	Lower Dibang Valley	262	7.14	7.14	42.86
Arunachal pradesh	Anjaw	263	14.29	35.71	35.71
Nagaland	Mon	264	15.38	15.38	0.00
Nagaland	Mokokchung	265	0.00	0.00	0.00
Nagaland	Zunheboto	266	16.67	0.00	0.00
Nagaland	Wokha	267	7.69	0.00	0.00
Nagaland	Dimapur	268	7.69	23.08	0.00
Nagaland	Phek	269	23.08	0.00	7.69
Nagaland	Tuensang	270	25.00	16.67	8.33
Nagaland	Longleng	271	0.00	7.69	0.00
Nagaland	Kiphire	272	30.77	38.46	0.00
Nagaland	Kohima	273	0.00	0.00	0.00
Nagaland	Peren	274	15.38	0.00	0.00
Manipur	Senapati	275	38.46	0.00	30.77
Manipur	Tamenglong	276	61.54	0.00	15.38
Manipur	Churachandpur	277	69.23	0.00	46.15
Manipur	Bishnupur	278	84.62	0.00	61.54
Manipur	Thoubal	279	100.00	0.00	30.77
Manipur	Imphal West	280	38.46	0.00	0.00
Manipur	Imphal East	281	100.00	0.00	46.15
Manipur	Ukhrul	282	46.15	0.00	15.38
Manipur	Chandel	283	92.31	0.00	53.85
Mizoram	Mamit	284	15.38	0.00	0.00
Mizoram	Kolasib	285	0.00	7.69	0.00
Mizoram	Aizawl	286	46.15	0.00	0.00
Mizoram	Champhai	287	0.00	0.00	0.00
Mizoram	Serchhip	288	8.33	0.00	25.00
Mizoram	Lunglei	289	16.67	0.00	8.33
Mizoram	Lawngtlai	290	15.38	0.00	30.77
Tripura	Dhalai	291	0.00	91.67	41.67
Meghalaya	South Garo Hills	292	25.00	0.00	16.67
Meghalaya	Ribhoi	293	50.00	16.67	33.33
Meghalaya	East Khasi Hills	294	23.08	0.00	7.69
Assam	Kokrajhar	295	23.08	38.46	7.69
Assam	Goalpara	296	15.38	76.92	15.38
Assam	Barpeta	297	25.00	58.33	8.33
Assam	Morigaon	298	61.54	53.85	15.38
Assam	Lakhimpur	299	8.33	8.33	25.00
Assam	Dhemaji	300	7.69	7.69	7.69
Assam	Tinsukia	301	8.33	75.00	50.00
Assam	Dibrugarh	302	0.00	0.00	41.67
Assam	Golaghat	303	0.00	50.00	50.00
Assam	Dima Hasao	304	0.00	0.00	30.77
Assam	Cachar	305	100.00	0.00	75.00
Assam	Karimganj	306	91.67	16.67	83.33
Assam	Hailakandi	307	84.62	30.77	61.54
Assam	Bongaigaon	308	7.69	84.62	46.15
Assam	Chirang	309	30.77	46.15	23.08
Assam	Kamrup	310	25.00	66.67	16.67
Assam	Kamrup Metropolitan	311	7.69	7.69	0.00
Assam	Nalbari	312	23.08	69.23	15.38
Assam	Baksa	313	8.33	8.33	50.00
Assam	Darrang	314	7.69	84.62	15.38
Assam	Udalguri	315	15.38	38.46	61.54
West bengal	Darjiling	316	38.46	69.23	7.69
West bengal	Jalpaiguri	317	41.67	75.00	8.33

West bengal	Koch Bihar	318	41.67	100.00	16.67
West bengal	Uttar Dinajpur	319	61.54	76.92	61.54
West bengal	Dakshin Dinajpur	320	30.77	84.62	15.38
West bengal	Maldah	321	61.54	100.00	38.46
West bengal	Murshidabad	322	91.67	100.00	25.00
West bengal	Birbhum	323	46.15	100.00	61.54
West bengal	Nadia	324	53.85	100.00	30.77
West bengal	North Twenty Four Parganas	325	16.67	66.67	16.67
West bengal	Hugli	326	53.85	100.00	53.85
West bengal	Bankura	327	58.33	100.00	41.67
West bengal	Puruliya	328	46.15	100.00	100.00
West bengal	South Twenty Four Parganas	329	33.33	91.67	41.67
West bengal	Paschim Medinipur	330	30.77	100.00	61.54
West bengal	Purba Medinipur	331	53.85	100.00	61.54
Jharkhand	Garhwa	332	28.57	100.00	0.00
Jharkhand	Chatra	333	0.00	92.86	21.43
Jharkhand	Kodarma	334	0.00	92.86	35.71
Jharkhand	Giridih	335	15.38	100.00	61.54
Jharkhand	Deoghar	336	0.00	92.31	23.08
Jharkhand	Godda	337	7.14	100.00	42.86
Jharkhand	Sahibganj	338	0.00	100.00	7.14
Jharkhand	Pakur	339	21.43	100.00	0.00
Jharkhand	Dhanbad	340	7.14	64.29	14.29
Jharkhand	Bokaro	341	15.38	84.62	7.69
Jharkhand	Lohardaga	342	21.43	92.86	0.00
Jharkhand	Purbi Singhbhum	343	0.00	28.57	7.14
Jharkhand	Palamu	344	21.43	85.71	14.29
Jharkhand	Latehar	345	0.00	92.86	21.43
Jharkhand	Hazaribagh	346	69.23	100.00	30.77
Jharkhand	Ramgarh	347	7.14	71.43	42.86
Jharkhand	Dumka	348	0.00	92.86	14.29
Jharkhand	Jamtara	349	15.38	100.00	61.54
Jharkhand	Ranchi	350	0.00	28.57	14.29
Jharkhand	Khunti	351	7.69	76.92	53.85
Jharkhand	Gumla	352	7.14	78.57	42.86
Jharkhand	Simdega	353	0.00	21.43	14.29
Jharkhand	Pashchimi Singhbhum	354	23.08	84.62	92.31
Jharkhand	Saraikela-Kharsawan	355	15.38	69.23	69.23
Odisha	Bargarh	356	16.67	41.67	33.33
Odisha	Jharsuguda	357	7.69	0.00	0.00
Odisha	Sambalpur	358	69.23	7.69	0.00
Odisha	Debagarh	359	46.15	23.08	46.15
Odisha	Sundargarh	360	15.38	15.38	0.00
Odisha	Kendujhar	361	61.54	69.23	76.92
Odisha	Mayurbhanj	362	46.15	100.00	38.46
Odisha	Baleshwar	363	84.62	38.46	92.31
Odisha	Bhadrak	364	61.54	7.69	61.54
Odisha	Kendrapara	365	61.54	0.00	69.23
Odisha	Cuttack	366	84.62	61.54	61.54
Odisha	Jajapur	367	46.15	30.77	84.62
Odisha	Dhenkanal	368	58.33	16.67	33.33
Odisha	Anugul	369	69.23	61.54	46.15
Odisha	Nayagarh	370	61.54	0.00	46.15
Odisha	Khordha	371	7.69	0.00	38.46
Odisha	Puri	372	46.15	0.00	7.69
Odisha	Ganjam	373	38.46	7.69	61.54
Odisha	Gajapati	374	30.77	100.00	23.08
Odisha	Kandhamal	375	58.33	25.00	66.67
Odisha	Baudh	376	84.62	61.54	53.85
Odisha	Subarnapur	377	69.23	7.69	15.38
Odisha	Balangir	378	15.38	15.38	23.08
Odisha	Nuapada	379	30.77	53.85	46.15

Odisha	Kalahandi	380	76.92	38.46	30.77
Odisha	Rayagada	381	61.54	100.00	46.15
Odisha	Nabarangapur	382	30.77	92.31	30.77
Odisha	Koraput	383	38.46	100.00	30.77
Odisha	Malkangiri	384	30.77	100.00	46.15
Chhattisgarh	Koriya	385	7.69	69.23	0.00
Chhattisgarh	Jashpur	386	8.33	83.33	25.00
Chhattisgarh	Raigarh	387	0.00	38.46	15.38
Chhattisgarh	Korba	388	7.69	84.62	15.38
Chhattisgarh	Janjgir-Champa	389	15.38	53.85	7.69
Chhattisgarh	Kabeerdham	390	0.00	100.00	16.67
Chhattisgarh	Rajnandgaon	391	53.85	23.08	23.08
Chhattisgarh	Mahasamund	392	15.38	92.31	30.77
Chhattisgarh	Dhamtari	393	8.33	33.33	16.67
Chhattisgarh	Uttar Bastar Kanker	394	8.33	33.33	50.00
Chhattisgarh	Narayanpur	395	53.85	61.54	30.77
Chhattisgarh	Bijapur	396	61.54	76.92	15.38
Madhya pradesh	Sheopur	397	61.54	92.31	69.23
Madhya pradesh	Morena	398	23.08	100.00	23.08
Madhya pradesh	Bhind	399	38.46	100.00	53.85
Madhya pradesh	Gwalior	400	23.08	46.15	15.38
Madhya pradesh	Datia	401	42.86	85.71	28.57
Madhya pradesh	Shivpuri	402	25.00	100.00	25.00
Madhya pradesh	Tikamgarh	403	16.67	100.00	16.67
Madhya pradesh	Chhatarpur	404	42.86	100.00	78.57
Madhya pradesh	Panna	405	50.00	100.00	50.00
Madhya pradesh	Sagar	406	42.86	28.57	71.43
Madhya pradesh	Damoh	407	35.71	100.00	42.86
Madhya pradesh	Satna	408	37.50	87.50	37.50
Madhya pradesh	Rewa	409	33.33	88.89	88.89
Madhya pradesh	Umaria	410	30.77	100.00	46.15
Madhya pradesh	Neemuch	411	50.00	92.86	78.57
Madhya pradesh	Mandsaur	412	21.43	85.71	42.86
Madhya pradesh	Ratlam	413	21.43	92.86	85.71
Madhya pradesh	Ujjain	414	0.00	84.62	76.92
Madhya pradesh	Dewas	415	42.86	100.00	92.86
Madhya pradesh	Dhar	416	71.43	100.00	57.14
Madhya pradesh	Indore	417	7.69	38.46	38.46
Madhya pradesh	Khargone (West Nimar)	418	0.00	92.31	76.92
Madhya pradesh	Barwani	419	53.85	92.31	84.62
Madhya pradesh	Rajgarh	420	50.00	100.00	100.00
Madhya pradesh	Vidisha	421	14.29	100.00	78.57
Madhya pradesh	Bhopal	422	0.00	40.00	40.00
Madhya pradesh	Sehore	423	35.71	92.86	100.00
Madhya pradesh	Raisen	424	33.33	100.00	66.67
Madhya pradesh	Betul	425	21.43	7.14	21.43
Madhya pradesh	Harda	426	21.43	92.86	50.00
Madhya pradesh	Hoshangabad	427	61.54	53.85	46.15
Madhya pradesh	Katni	428	50.00	100.00	62.50
Madhya pradesh	Jabalpur	429	25.00	100.00	25.00
Madhya pradesh	Narsimhapur	430	7.69	38.46	30.77
Madhya pradesh	Dindori	431	21.43	92.86	42.86
Madhya pradesh	Mandla	432	21.43	71.43	28.57
Madhya pradesh	Chhindwara	433	15.38	76.92	69.23
Madhya pradesh	Seoni	434	30.77	76.92	7.69
Madhya pradesh	Balaghat	435	28.57	0.00	7.14
Madhya pradesh	Guna	436	25.00	100.00	75.00
Madhya pradesh	Ashoknagar	437	27.27	100.00	27.27
Madhya pradesh	Shahdol	438	0.00	100.00	0.00
Madhya pradesh	Anuppur	439	21.43	100.00	0.00
Madhya pradesh	Singrauli	440	16.67	100.00	16.67
Madhya pradesh	Jhabua	441	69.23	100.00	69.23
Madhya pradesh	Alirajpur	442	7.69	100.00	53.85
Madhya pradesh	Khandwa (East Nimar)	443	50.00	100.00	50.00

Madhya pradesh	Burhanpur	444	14.29	28.57	57.14
Gujarat	Kachchh	445	0.00	38.46	30.77
Gujarat	Banas Kantha	446	69.23	69.23	100.00
Gujarat	Patan	447	15.38	76.92	46.15
Gujarat	Mahesana	448	0.00	8.33	8.33
Gujarat	Gandhinagar	449	25.00	91.67	58.33
Gujarat	Porbandar	450	23.08	0.00	30.77
Gujarat	Anand	451	69.23	30.77	23.08
Gujarat	Dohad	452	50.00	100.00	33.33
Gujarat	Narmada	453	50.00	33.33	25.00
Gujarat	Bharuch	454	8.33	25.00	33.33
Gujarat	The Dangs	455	7.69	38.46	7.69
Gujarat	Navsari	456	15.38	7.69	30.77
Gujarat	Valsad	457	15.38	7.69	7.69
Gujarat	Tapi	458	0.00	16.67	8.33
Dadra & nagar haveli and daman & diu	Daman	459	0.00	15.38	15.38
Dadra & nagar haveli and daman & diu	Dadra & Nagar Haveli	460	0.00	38.46	38.46
Maharashtra	Nandurbar	461	69.23	100.00	92.31
Maharashtra	Dhule	462	84.62	53.85	100.00
Maharashtra	Jalgaon	463	84.62	46.15	61.54
Maharashtra	Buldana	464	30.77	76.92	53.85
Maharashtra	Akola	465	53.85	23.08	53.85
Maharashtra	Washim	466	38.46	61.54	61.54
Maharashtra	Amravati	467	84.62	0.00	53.85
Maharashtra	Wardha	468	58.33	0.00	41.67
Maharashtra	Nagpur	469	23.08	0.00	46.15
Maharashtra	Bhandara	470	58.33	0.00	75.00
Maharashtra	Gondiya	471	46.15	0.00	15.38
Maharashtra	Gadchiroli	472	61.54	0.00	76.92
Maharashtra	Chandrapur	473	23.08	0.00	69.23
Maharashtra	Yavatmal	474	46.15	15.38	61.54
Maharashtra	Nanded	475	58.33	100.00	83.33
Maharashtra	Hingoli	476	69.23	84.62	84.62
Maharashtra	Parbhani	477	76.92	92.31	69.23
Maharashtra	Jalna	478	75.00	91.67	100.00
Maharashtra	Nashik	479	58.33	8.33	75.00
Maharashtra	Mumbai Suburban	480	0.00	0.00	8.33
Maharashtra	Mumbai	481	7.69	0.00	7.69
Maharashtra	Raigarh	482	38.46	23.08	23.08
Maharashtra	Pune	483	38.46	7.69	15.38
Maharashtra	Ahmadnagar	484	91.67	75.00	83.33
Maharashtra	Bid	485	75.00	83.33	100.00
Maharashtra	Latur	486	75.00	91.67	75.00
Maharashtra	Osmanabad	487	46.15	38.46	23.08
Maharashtra	Solapur	488	41.67	91.67	41.67
Maharashtra	Satara	489	58.33	50.00	41.67
Maharashtra	Ratnagiri	490	46.15	0.00	46.15
Maharashtra	Sindhudurg	491	8.33	0.00	66.67
Maharashtra	Kolhapur	492	23.08	7.69	15.38
Maharashtra	Sangli	493	33.33	16.67	25.00
Karnataka	Belgaum	494	100.00	58.33	41.67
Karnataka	Bagalkot	495	100.00	92.31	61.54
Karnataka	Bijapur	496	100.00	92.31	92.31
Karnataka	Bidar	497	75.00	75.00	66.67
Karnataka	Raichur	498	91.67	100.00	91.67
Karnataka	Koppal	499	84.62	61.54	61.54
Karnataka	Gadag	500	100.00	61.54	100.00
Karnataka	Dharwad	501	91.67	58.33	50.00
Karnataka	Uttara Kannada	502	100.00	7.69	76.92
Karnataka	Haveri	503	92.31	23.08	100.00
Karnataka	Bellary	504	100.00	69.23	92.31
Karnataka	Chitradurga	505	83.33	50.00	58.33
Karnataka	Davanagere	506	100.00	16.67	66.67
Karnataka	Shimoga	507	92.31	0.00	38.46

Karnataka	Chikmagalur	508	100.00	0.00	69.23
Karnataka	Tumkur	509	92.31	0.00	61.54
Karnataka	Bangalore	510	100.00	0.00	41.67
Karnataka	Mandya	511	100.00	7.69	38.46
Karnataka	Hassan	512	100.00	0.00	46.15
Karnataka	Dakshina Kannada	513	84.62	0.00	76.92
Karnataka	Kodagu	514	92.31	0.00	46.15
Karnataka	Mysore	515	92.31	53.85	61.54
Karnataka	Chamarajanagar	516	100.00	61.54	76.92
Karnataka	Gulbarga	517	100.00	58.33	100.00
Karnataka	Yadgir	518	83.33	91.67	100.00
Karnataka	Kolar	519	76.92	23.08	84.62
Karnataka	Chikkaballapura	520	100.00	46.15	46.15
Karnataka	Bangalore Rural	521	75.00	0.00	41.67
Karnataka	Ramanagara	522	100.00	0.00	92.31
Goa	North Goa	523	15.38	0.00	30.77
Goa	South Goa	524	7.69	0.00	0.00
Kerala	Wayanad	525	53.85	0.00	61.54
Kerala	Kozhikode	526	50.00	0.00	58.33
Kerala	Malappuram	527	38.46	0.00	53.85
Kerala	Palakkad	528	23.08	0.00	15.38
Kerala	Thrissur	529	16.67	0.00	100.00
Kerala	Ernakulam	530	30.77	0.00	0.00
Kerala	Idukki	531	46.15	0.00	15.38
Kerala	Kottayam	532	30.77	0.00	46.15
Kerala	Pathanamthitta	533	76.92	0.00	23.08
Kerala	Kollam	534	76.92	0.00	30.77
Kerala	Thiruvananthapuram	535	53.85	0.00	46.15
Tamil nadu	Chennai	536	100.00	0.00	23.08
Tamil nadu	Kancheepuram	537	100.00	0.00	69.23
Tamil nadu	Vellore	538	100.00	8.33	25.00
Tamil nadu	Tiruvannamalai	539	100.00	0.00	41.67
Tamil nadu	Viluppuram	540	100.00	7.69	69.23
Tamil nadu	Salem	541	100.00	0.00	50.00
Tamil nadu	Namakkal	542	100.00	0.00	23.08
Tamil nadu	Erode	543	100.00	7.69	15.38
Tamil nadu	The Nilgiris	544	100.00	0.00	0.00
Tamil nadu	Dindigul	545	100.00	0.00	53.85
Tamil nadu	Karur	546	100.00	15.38	53.85
Tamil nadu	Tiruchirappalli	547	100.00	0.00	41.67
Tamil nadu	Perambalur	548	100.00	0.00	30.77
Tamil nadu	Ariyalur	549	100.00	0.00	38.46
Tamil nadu	Cuddalore	550	100.00	0.00	16.67
Tamil nadu	Nagapattinam	551	100.00	0.00	69.23
Tamil nadu	Thiruvarur	552	92.31	0.00	30.77
Tamil nadu	Thanjavur	553	100.00	0.00	69.23
Tamil nadu	Pudukkottai	554	100.00	0.00	75.00
Tamil nadu	Sivaganga	555	100.00	0.00	23.08
Tamil nadu	Madurai	556	100.00	0.00	7.69
Tamil nadu	Theni	557	92.31	0.00	53.85
Tamil nadu	Virudhunagar	558	100.00	0.00	33.33
Tamil nadu	Thoothukkudi	559	100.00	0.00	16.67
Tamil nadu	Tirunelveli	560	100.00	0.00	58.33
Tamil nadu	Kanniyakumari	561	100.00	0.00	7.69
Tamil nadu	Dharmapuri	562	100.00	0.00	0.00
Tamil nadu	Krishnagiri	563	84.62	23.08	0.00
Tamil nadu	Coimbatore	564	76.92	0.00	7.69
Tamil nadu	Tiruppur	565	100.00	0.00	0.00
Puducherry	Yanam	566	84.62	0.00	84.62
Puducherry	Puducherry	567	92.31	0.00	46.15
Arunachal pradesh	East Siang	568	69.23	38.46	46.15
Arunachal pradesh	Kra Daadi	569	25.00	37.50	87.50
Arunachal pradesh	Kurung Kumey	570	0.00	0.00	28.57
Arunachal pradesh	Lohit	571	7.14	42.86	35.71
Arunachal pradesh	Longding	572	15.38	7.69	61.54

Arunachal pradesh	Namsai	573	0.00	53.85	53.85
Arunachal pradesh	Siang	574	28.57	7.14	57.14
Arunachal pradesh	Tirap	575	14.29	0.00	64.29
Arunachal pradesh	West Siang	576	30.77	53.85	46.15
Assam	Biswanath	577	7.69	53.85	53.85
Assam	Charaideo	578	0.00	53.85	38.46
Assam	Dhubri	579	30.77	100.00	23.08
Assam	Hojai	580	8.33	50.00	8.33
Assam	Jorhat	581	0.00	46.15	30.77
Assam	Karbi Anglong	582	15.38	46.15	15.38
Assam	Majuli	583	0.00	23.08	23.08
Assam	Nagaon	584	15.38	69.23	53.85
Assam	Sivasagar	585	15.38	53.85	23.08
Assam	Sonitpur	586	7.69	30.77	7.69
Assam	South Salmara Mancachar	587	38.46	84.62	15.38
Assam	West Karbi Anglong	588	7.69	30.77	53.85
Chhattisgarh	Balod	589	7.69	61.54	23.08
Chhattisgarh	Baloda Bazar	590	0.00	84.62	15.38
Uttar pradesh	Balrampur	591	85.71	92.86	85.71
Chhattisgarh	Bastar	592	46.15	69.23	53.85
Chhattisgarh	Bemetara	593	8.33	100.00	25.00
Chhattisgarh	Bilaspur	594	15.38	92.31	23.08
Chhattisgarh	Dantewada	595	30.77	84.62	53.85
Chhattisgarh	Durg	596	7.69	7.69	7.69
Chhattisgarh	Gariyaband	597	15.38	15.38	23.08
Chhattisgarh	Kodagaon	598	33.33	75.00	16.67
Chhattisgarh	Mungeli	599	16.67	100.00	8.33
Chhattisgarh	Raipur	600	23.08	76.92	7.69
Chhattisgarh	Sukma	601	23.08	76.92	30.77
Chhattisgarh	Surguja	602	15.38	53.85	23.08
Nct of delhi	Central	603	9.09	0.00	90.91
Nct of delhi	East	604	0.00	23.08	30.77
Nct of delhi	New Delhi	605	0.00	23.08	53.85
Nct of delhi	North	606	7.69	7.69	69.23
Nct of delhi	North East	607	0.00	16.67	25.00
Nct of delhi	North West	608	0.00	16.67	16.67
Nct of delhi	Shahdara	609	0.00	0.00	53.85
Nct of delhi	South	610	0.00	0.00	46.15
Nct of delhi	South East	611	0.00	0.00	0.00
Nct of delhi	South West	612	8.33	0.00	25.00
Nct of delhi	West	613	0.00	30.77	46.15
Gujarat	Ahmadabad	614	0.00	0.00	38.46
Gujarat	Aravali	615	30.77	84.62	30.77
Gujarat	Bhavnagar	616	0.00	33.33	33.33
Gujarat	Botad	617	7.69	69.23	0.00
Gujarat	Chhota Udaipur	618	0.00	92.31	23.08
Gujarat	Devbhumi Dwarka	619	38.46	46.15	69.23
Gujarat	Gir Somnath	620	0.00	58.33	41.67
Gujarat	Jamnagar	621	38.46	7.69	46.15
Gujarat	Junagadh	622	38.46	0.00	84.62
Gujarat	Kheda	623	46.15	46.15	61.54
Gujarat	Mahisagar	624	8.33	16.67	83.33
Gujarat	Morbi	625	53.85	0.00	46.15
Gujarat	Panch Mahals	626	15.38	61.54	0.00
Gujarat	Rajkot	627	0.00	0.00	0.00
Gujarat	Sabar Kantha	628	30.77	53.85	23.08
Gujarat	Surendranagar	629	30.77	7.69	46.15
Gujarat	Vadodara	630	30.77	23.08	53.85
Haryana	Bhiwani	631	7.69	38.46	46.15
Haryana	Charkhi Dadri	632	0.00	0.00	16.67
Madhya pradesh	Agar Malwa	633	28.57	100.00	35.71
Madhya pradesh	Shajapur	634	61.54	100.00	76.92
Maharashtra	Palghar	635	30.77	46.15	7.69
Maharashtra	Thane	636	7.69	7.69	23.08
Meghalaya	East Garo Hills	637	84.62	23.08	38.46

Meghalaya	East Jantia Hills	638	15.38	61.54	7.69
Meghalaya	North Garo Hills	639	76.92	46.15	69.23
Meghalaya	South West Garo Hills	640	53.85	0.00	46.15
Meghalaya	South West Khasi Hills	641	30.77	7.69	0.00
Meghalaya	West Garo Hills	642	46.15	0.00	15.38
Meghalaya	West Jaintia Hills	643	33.33	66.67	16.67
Meghalaya	West Khasi Hills	644	30.77	38.46	0.00
Punjab	Fazilka	645	15.38	15.38	30.77
Punjab	Firozpur	646	8.33	0.00	0.00
Punjab	Gurdaspur	647	15.38	0.00	7.69
Punjab	Pathankot	648	8.33	0.00	16.67
Telangana	Adilabad	649	100.00	84.62	92.31
Telangana	Bhadradri Kothagudem	650	100.00	69.23	84.62
Telangana	Hyderabad	651	92.31	0.00	38.46
Telangana	Jagitial	652	84.62	100.00	61.54
Telangana	Jangoan	653	100.00	100.00	100.00
Telangana	Jayashankar Bhupalapally	654	100.00	100.00	46.15
Telangana	Jogulamba Gadwal	655	91.67	100.00	100.00
Telangana	Kamareddy	656	100.00	100.00	100.00
Telangana	Karimnagar	657	92.31	92.31	100.00
Telangana	Khammam	658	92.31	84.62	69.23
Telangana	Komaram Bheem Asifabad	659	92.31	100.00	76.92
Telangana	Mahabubabad	660	100.00	100.00	91.67
Telangana	Mahabubnagar	661	100.00	100.00	69.23
Telangana	Mancherial	662	100.00	91.67	50.00
Telangana	Medak	663	92.31	100.00	92.31
Telangana	Medchal-Malkajgiri	664	91.67	16.67	75.00
Telangana	Nagarkurnool	665	91.67	100.00	100.00
Telangana	Nalgonda	666	100.00	92.31	84.62
Telangana	Nirmal	667	92.31	92.31	61.54
Telangana	Nizamabad	668	100.00	69.23	92.31
Telangana	Peddapalli	669	84.62	100.00	76.92
Telangana	Rajanna Sircilla	670	100.00	61.54	38.46
Telangana	Ranga Reddy	671	100.00	58.33	66.67
Telangana	Sangareddy	672	92.31	92.31	100.00
Telangana	Siddipet	673	100.00	53.85	92.31
Telangana	Suryapet	674	92.31	92.31	84.62
Telangana	Vikarabad	675	100.00	100.00	100.00
Telangana	Wanaparthy	676	100.00	100.00	84.62
Telangana	Warangal Rural	677	92.31	100.00	76.92
Telangana	Warangal Urban	678	84.62	53.85	69.23
Telangana	Yadadri Bhuvanagiri	679	100.00	84.62	53.85
Tripura	Gomati	680	25.00	91.67	33.33
Tripura	Khowai	681	7.69	30.77	15.38
Tripura	North Tripura	682	53.85	76.92	15.38
Tripura	South Tripura	683	8.33	100.00	33.33
Tripura	Unakoti	684	53.85	61.54	38.46
Tripura	West Tripura	685	15.38	61.54	7.69
Uttar pradesh	Amethi	686	14.29	78.57	0.00
Uttar pradesh	Budaun	687	28.57	100.00	57.14
Uttar pradesh	Ghaziabad	688	0.00	0.00	33.33
Uttar pradesh	Hapur	689	30.77	38.46	23.08
Uttar pradesh	Moradabad	690	46.15	69.23	30.77
Uttar pradesh	Muzaffarnagar	691	7.14	50.00	35.71
Uttar pradesh	Rae Bareli	692	76.92	92.31	69.23
Uttar pradesh	Sambhal	693	42.86	100.00	64.29
Uttar pradesh	Shamli	694	7.69	76.92	38.46
Uttar pradesh	Sultanpur	695	57.14	42.86	28.57
West bengal	Paschim Barddhaman	696	25.00	100.00	75.00

West bengal	Purba Barddhaman	697	46.15	100.00	38.46
Tamil nadu	Thiruvallur	698	100.00	0.00	69.23
Gujarat	Surat	699	0.00	0.00	15.38
Gujarat	Amreli	700	0.00	25.00	0.00
Chhattisgarh	Surajpur	701	0.00	100.00	8.33
Madhya pradesh	Sidhi	702	46.15	92.31	61.54
Uttar pradesh	Kheri	703	78.57	100.00	35.71
Tripura	Sepahijala	704	75.00	100.00	41.67
Karnataka	Udupi	705	69.23	0.00	61.54
Kerala	Kasaragod	706	15.38	0.00	15.38
Lakshadweep	Lakshadweep	707	30.77	0.00	15.38
Kerala	Kannur	708	7.69	0.00	0.00
Kerala	Alappuzha	709	30.77	0.00	23.08
Mizoram	Saiha	710	53.85	0.00	7.69
Andaman & nicobar islands	South Andaman	711	0.00	0.00	0.00
Tamil nadu	Ramanathapuram	712	100.00	0.00	61.54
Andaman & nicobar islands	Nicobars	713	7.69	0.00	38.46
Puducherry	Karaikal	714	66.67	0.00	58.33
West bengal	Haora	715	30.77	53.85	38.46
Odisha	Jagatsinghapur	716	58.33	0.00	91.67
Andaman & nicobar islands	North & Middle Andaman	717	0.00	25.00	0.00
Ladakh	Kargil	718	53.85	0.00	92.31
Bihar	Aurangabad	720	38.46	100.00	69.23
Maharashtra	Aurangabad	721	50.00	58.33	58.33
Dadra & nagar haveli and daman & diu	Diu	722	0.00	0.00	23.08
Puducherry	Mahe	723	0.00	0.00	15.38

Note.

- Total 9,104 communities, 720 districts, and 36 states are included.
- Under-empowered communities are defined as those with a prevalence of low level of empowerment equal to or higher than the national mean of low level of empowerment.
